# Academic achievement map: a clear cognitive route and a promising motor path

**DOI:** 10.1016/j.jped.2026.101611

**Published:** 2026-09-23

**Authors:** Valter Fernandes, Núbia Gabriele de Souza Nunes, Natália Mota, Sidarta Ribeiro, Andrea Deslandes

**Affiliations:** a Universidade Federal do Rio de Janeiro (UFRJ), Rio de Janeiro, RJ, Brazil; b Research Department Mobile Brain, Universidade Federal do Rio de Janeiro (UFRJ), Rio de Janeiro, RJ, Brazil; c Brain Institute, Universidade Federal do Rio Grande do Norte (UFRN), Natal, RN, Brazil; d Center for Strategic Studies, Fundação Oswaldo Cruz (FIOCRUZ), Rio de Janeiro, RJ, Brazil

**Keywords:** Academic performance, Executive functions, Eye-hand coordination, Working memory, Touch test disc

## Abstract

**Objective:**

This study investigates how executive functions, motor coordination, and oral discourse are related to academic performance in children from public schools in Rio de Janeiro.

**Method:**

This longitudinal study assessed 75 children at the beginning (T1) and at the end (T2) of a school semester. Working memory was measured by the *Digit Span* test, eye-hand coordination by the Touch Test Disc (TTD), and oral discourse by non-semantic graph metrics (SpeechGraphs). Academic performance was assessed in Portuguese and Mathematics.

**Results:**

Digit Span and TTD showed the strongest correlations with academic performance at T2, while graph metrics obtained from oral discourse were not significant predictors. Digit Span correlated positively with Portuguese (r = 0.505; p < 0.001) and Mathematics (r = 0.388; p = 0.002), while TTD correlated negatively with Portuguese (r = -0.453; p < 0.001) and Mathematics (r = -0.398; p = 0.001), i.e. better motor performance was associated with higher grades. Multiple regression analyses showed that Digit Span predicted Portuguese (β = 0.492; p < 0.001, 24.2% of variance). For Mathematics, both age (β = -0.501; p < 0.001) and Digit Span performance (β = 0.413; p = 0.002) were significant predictors.

**Conclusion:**

Working memory is a predictor of academic performance in both Portuguese and Mathematics, and eye-hand coordination also shows significant correlations with school grades, suggesting a relevant relationship between motor skills and learning. These findings highlight the importance of taking an integrated approach to cognitive and motor capacities in the educational context.

## Introduction

Academic performance is influenced by an interaction between cognitive, linguistic, socio-emotional, and motor aspects from early childhood onward [1]. In elementary school, children face the challenge of learning how to read, write, and perform basic mathematical operations, tasks that stimulate and demand the development of cognitive skills known as executive functions (EFs). A widely adopted theoretical model states that executive functions (EFs) consist of three core components that are distinct yet intertwined: working memory, inhibitory control, and cognitive flexibility [2]. The ability to hold and manipulate information mentally characterizes the working memory construct, while inhibitory control refers to the capacity to suppress automatic or prepotent responses. Derived from those abilities, cognitive flexibility is expressed in the ability to switch between previously planned actions [2]. Working memory has been identified as a crucial component of academic success. It plays a role in both text comprehension and mathematical problem-solving by enabling children to mentally maintain and manipulate information [3]. On the other hand, inhibitory control supports focused attention in the classroom by enabling children to filter out distractions and engage in behaviors directed toward academic goals [3]. Despite these highly interrelated developmental trajectories between EFs and academic achievement, school success depends on a broader range of aspects, from socioeconomic status to several social, emotional and cognitive skills [4].

Beyond executive functions, the ability to produce coherent and well-structured oral discourse also proves to be an important element of learning, as narrative production reflects complex skills of thought organization fundamental for written expression and reading comprehension [5]. The analysis of non-semantic graphs provides an objective quantification of discourse structure, capturing elements like connectivity across words, found to be related to reading performance in children [6].

The development of both the motor and cognitive domains is interconnected, with both aspects evolving in an integrated manner [7]. However, the relationship between children's gross motor skills and cognition appears to be related to a common underlying process of executive EFs [8]. Investigating this specific neural connection, neuroimaging research has revealed that practicing complex, goal-directed motor activities can alter the structure and function of brain regions essential for EFs and motor control, such as the dorsolateral prefrontal cortex, the anterior cingulate cortex, and the cerebellum [9,10]. This co-activation suggests that motor coordination and higher-order cognitive processes share common neural underpinnings, which may explain their frequent correlation in behavioral tasks.

This neurobiological connection can be enhanced by engagement in physical activities, which is facilitated by a higher level of motor competence, [11] helping to explain why physical exercise interventions can have positive effects on both physical fitness and academic performance [12]. In this context, the development of fine motor skills and eye-hand coordination stands out as an essential foundation for literacy, as fundamental tasks such as pencil handling, letter tracing, and spatial organization on paper depend on this competence [13]. Eye-hand coordination, which involves integrating visual information with hand motor control, is essential for any precise activity, such as writing, drawing, manipulating objects, throwing a ball or hitting it with a racket [14]. Similarly, a recent study showed a strong relationship between motor competence and academic achievement, which is mediated by fitness skills and executive functions [15]. Therefore, promoting motor-enriched interventions may be a feasible way to improve academic success. However, difficulties in motor development can be an early sign of potential learning gaps [16].

Complex motor skills, such as hand-eye coordination, are typically evaluated using the Touch Test Disc (TTD) and have been identified as predictors of improved academic performance and executive function (EF) skills [17,18]. However, the relationship between these skills and academic performance needs to be investigated in different socioeconomic contexts. In this regard, the literature lacks longitudinal studies integrating measures of specific executive function components, oral discourse, and motor coordination to predict academic performance. This gap is particularly evident in samples from Brazilian public schools, where socioeconomic vulnerabilities significantly impact academic achievement [19]. To address this limitation, the present study investigates the hypothesis that higher scores on working memory, inhibitory control, motor coordination, agility, and oral discourse organization tests predict better academic performance in elementary school children.

## Method

### Participants

This prospective longitudinal observational study is part of a larger research project entitled "Motiveducação - Physical exercise for child development". The sample consisted of subjects enrolled in and assigned to the control group of the project during 2024 and 2025. The participants in this analysis represent the control cohort of a broader randomized controlled trial, consisting exclusively of children who did not receive any extracurricular physical exercise intervention during the study period, maintaining only their regular school activities. Recruitment took place in two municipal public schools under the 7th Regional Education Coordination (CRE), located in Freguesia de Jacarepaguá, Rio de Janeiro. At the beginning of the school year, extracurricular activities in Capoeira and Athletics were offered to students from 3rd to 9th grade, as part of the chronic exercise intervention axis of the main project. The inclusion criteria were regular enrolment in an eligible year group, an interest in one of the offered sports, and a declaration of adequate health to participate. Exclusion criteria defined by the Motiveducação project included uncorrected hearing or visual impairment, or neurodevelopmental disorders that would prevent participation in assessments.

Legal guardians were contacted, received detailed information about study procedures, and signed the Informed Consent Form (ICF), while children signed the Assent Form. All participants completed a registration and anamnesis form. After consent and baseline assessments (pre-test), children were randomly allocated to the group that did not receive extracurricular physical exercise intervention during the first semester, maintaining only regular school activities. The study was approved by the Research Ethics Committee of IPUB/UFRJ (7.325.472).

### Procedures

According to the study design, two assessment phases were conducted at the beginning of the school year (T1, February — March) and at the end of the first semester (T2, June — July). The longitudinal design was employed to capture the predictive value of baseline cognitive, motor, and linguistic measures on subsequent academic achievement, rather than to analyze within-subject changes over time. Specifically in the initial assessments, academic performance was evaluated based on the grades from the last bimonthly of the previous year. All other assessments (cognitive, motor, and discourse) were carried out at both T1 and T2. Assessments were conducted individually within the school environment by a trained team, following standardized protocols. Evaluation occurred over three sessions, lasting approximately 3 — 4 h per child to prevent fatigue. The first session (∼60 min) included anthropometric measures and the Touch Test Disc. The second session (∼90 min) included cardiorespiratory fitness and agility tests. The third session (60—90 min) included oral discourse collection and executive function tests (Digit Span and Hearts and Flowers).

#### Sample size and missing data

A formal a priori sample size calculation was not performed for this specific analysis, as the sample size was constrained by the recruitment capacity within the participating schools and the number of children allocated to the control group of the parent project. From an initial pool of 158 children allocated to the control group, 83 were excluded from the present analysis: 4 due to a confirmed diagnosis of neurodevelopmental disorders (a pre-established exclusion criterion), and 79 due to missing data, such as academic grades in Portuguese and Mathematics at Time 2 (T2) or incomplete results in the executive function tests. This substantial loss to follow-up (∼50%; 83 out of 158 children) represents a limitation, and its potential for introducing selection bias must be acknowledged. Specifically, children with missing data may have differed systematically from those retained, which could affect the generalizability of the present findings. The final study sample thus consisted of 75 children (38 boys and 37 girls), with a mean age of 9.84 years (SD = 1.77). No significant differences were observed between sexes in the variables investigated.

### Instruments

The Touch Test Disc (TTD) assessed hand-eye coordination, motor planning, and processing speed [20]. The test was performed using a rectangular wooden board (120 × 60 cm). A central rectangle (10 × 20 cm) and a 20 cm circle on each side, spaced 5 cm apart, were positioned on the board. The participant kept the non-dominant hand in the central rectangle and used the dominant hand to touch the opposite circle, crossing one arm over the other, completing a cycle. Each attempt consisted of 25 correct cycles, and the minimum time to complete three attempts was recorded.

Agility was assessed using the square test. A 4 × 4 m square was marked with tape, with cones at each corner. The student stood beside a cone and, upon the evaluator's signal, ran as fast as possible, touching each cone with one hand, crossing two diagonals and two straight paths until all cones were reached. Two trials were performed, and the shortest time was recorded.

Identification and anthropometric data were collected using a registration and anamnesis form. Data recorded included age, sex, self-declared race/color, and residence. Anthropometric measures included weight and seated height to calculate Body Mass Index and biological maturity estimated using Peak Height Velocity (PHV), following Mirwald et al. [21].

Oral narratives (image description) lasting 30 s to 3 min were recorded. Transcriptions were later analyzed using the SpeechGraphs software [22]. To account for non-pathological verbosity differences across subjects, graph analysis used a sliding window with a fixed length of 30 words, with 50% overlap between consecutive windows (i.e., steps of 15 words). Average graph attributes were calculated from all windows for each participant. The following four graph-based metrics were extracted: The Largest Strongly Connected Component (LSC), which reflects the global connectivity of discourse; the Average Shortest Path (ASP), which measures the efficiency of semantic connections; the number of Nodes (NO), which indicates lexical diversity; and the Repeated Edges (RE), which assesses the recurrence of words.

Executive functions were assessed using the Hearts and Flowers test, [23] which measures inhibitory control, and the Digit Span test, [24] which measures verbal working memory. Both tests were administered via tablet using the Psytoolkit platform and took approximately 20 min to complete. In the Hearts and Flowers task, participants are given congruent and incongruent commands. When a heart appears, the child must tap the same side of the screen. When a flower appears, the child must tap the opposite side. The task includes three phases: blocks with only hearts, blocks with only flowers, and mixed blocks. In this study, the authors used the mixed block phase and recorded reaction time and accuracy to calculate the cost score for correct responses (HFT) using the following formula: (mean reaction time/percentage of correct responses) / 100. A lower cost score indicates better executive function performance [18]. The Digit Span task assesses verbal working memory through two conditions: Forward and Backward. In the forward condition, the child hears a sequence of numbers and must repeat them in the same order, measuring attention and short-term auditory memory. In the backward condition, the child must repeat the sequence in reverse order, which requires mental manipulation of information and is considered a purer measure of working memory. The total score is the sum of the maximum sequence length correctly repeated in both conditions, with a limit of two errors. A higher score indicates greater working memory capacity [24]. Academic performance was evaluated using school grades in Portuguese Language and Mathematics. First-term grades of 2025 (T1) and second-term grades (T2) were collected.

### Statistical analysis

Descriptive analyses (means, standard deviations) were conducted to characterize the sample. Data normality was verified using the Kolmogorov-Smirnov test. Mean differences between sexes were examined using Student's *t*-test for independent samples or the Mann-Whitney test, according to normality. Pearson and Spearman correlations, controlled for sex and age, were performed among all study variables with Bonferroni correction. Multiple linear regression analyses were conducted to investigate the predictive value of executive functions, physical fitness, and discourse measures for academic performance at T2. The significance level was set at p ≤ 0.05. Analyses were conducted using JASP software (version 0.18.1).

## Results

The demographic, cognitive, and motor characteristics of the final sample are described in Table 1. From an initial pool of 158 children allocated to the control group, 83 were excluded from the present analysis: 4 due to a diagnosis of neurodevelopmental disorders (a pre-established exclusion criterion), and 79 due to missing data, such as academic grades in Portuguese and Mathematics at Time 2 (T2) or results in the executive function tests. The final study sample thus consisted of 75 children (38 boys and 37 girls), with a mean age of 9.84 years (SD = 1.77). No significant differences were observed between sexes in the variables investigated.

**Table 1:** Descriptive statistics of the sample by sex.

	Male		Female		Total			
	(n = 38)		(n = 37)		(n = 75)			
	M	(DP)	M	(DP)	M	(DP)	F	p
Age (years)	10.00	1.68	9.68	1.87	9.84	1.77	−0.791	0.432
Race (% White / % Non-White)	32.6% / 67.4%	–	30.0% / 70.0%	–	31.5% / 68.5%	–	–	–
Height (cm)	142.91	14.01	140.02	11.97	141.46	13.06	−0.810	0.422
Weight (kg)	37.44	15.23	38.58	16.31	38.01	15.77	0.305	0.759
PHV (cm/year)	14.04	4.56	11.83	0.88	12.94	3.36	−1.852	0.113
BMI (kg/m^2^)	18.05	4.08	19.86	6.08	18.96	5.18	1081	0.285
Digit Span (score)	5.94	2.24	5.90	1.89	5.92	2.06	−0.071	0.949
Hearts and Flowers (score)	14.53	5.34	16.27	9.04	15.40	7.39	52946	0.348
Touch Test Disc (seconds)	21.23	3.98	22.14	4.92	21.69	4.45	0.826	0.412
Agility (seconds)	7.80	1.12	7.63	0.72	7.72	0.93	0.102	0.919
Mathematics T1(score)	5.77	2.65	6.61	1.75	6.19	2.27	1.584	0.118
Portuguese T1 (score)	6.33	2.40	6.92	1.61	6.62	2.06	1.214	0.229
Mathematics T2(score)	5.83	2.31	5.66	2.26	5.74	2.28	−0.511	0.611
Portuguese T2 (score)	6.25	2.17	6.49	2.00	6.37	2.08	0.487	0.628
Oral Discurse – ASP (score)	4.00	0.38	4.13	0.58	4.06	0.49	0.988	0.328
Oral Discurse – LSC (score)	16.90	2.04	16.43	2.06	16.67	2.05	−0.311	0.757
Oral Discurse – NO (score)	23.32	1.17	23.45	1.57	23.39	1.37	0.334	0.725
Oral Discurse – RE (score)	1.31	0.71	1.30	0.91	1.31	0.81	−0.061	0.952

* Note: M, Mean; SD, Standard Deviation; F, Student's *t*-test statistic; p, p-value. T1, first assessment time (end of 2024); T2, second assessment time (beginning of 2025). Oral Discourse metrics: ASP, Average Shortest Path; LSC, Largest Strongly Connected Component; NO, Number of Nodes; RE, Repeated Edges. PHV, Peak Height Velocity; BMI, Body Mass Index.

Figure 1 presents the heat map of correlations between the study variables. Digit Span showed significant positive correlations with Portuguese T2 (r = 0.505; p < 0.001) and Mathematics T2 (r = 0.388; p = 0.002). The Touch Test Disc showed significant negative correlations with Portuguese T2 (r = −0.453; p < 0.001) and Mathematics T2 (r = −0.398; p = 0.001), indicating that shorter execution time (better eye-hand coordination) is associated with better academic grades. The remaining variables, including the oral discourse metrics (ASP, LSC, NO, RE), the Hearts and Flowers test, the horizontal jump, agility, BMI, and PHV, did not show significant correlations with academic performance in Portuguese and Mathematics at T2 after the Bonferroni correction.

**Figure 1:**
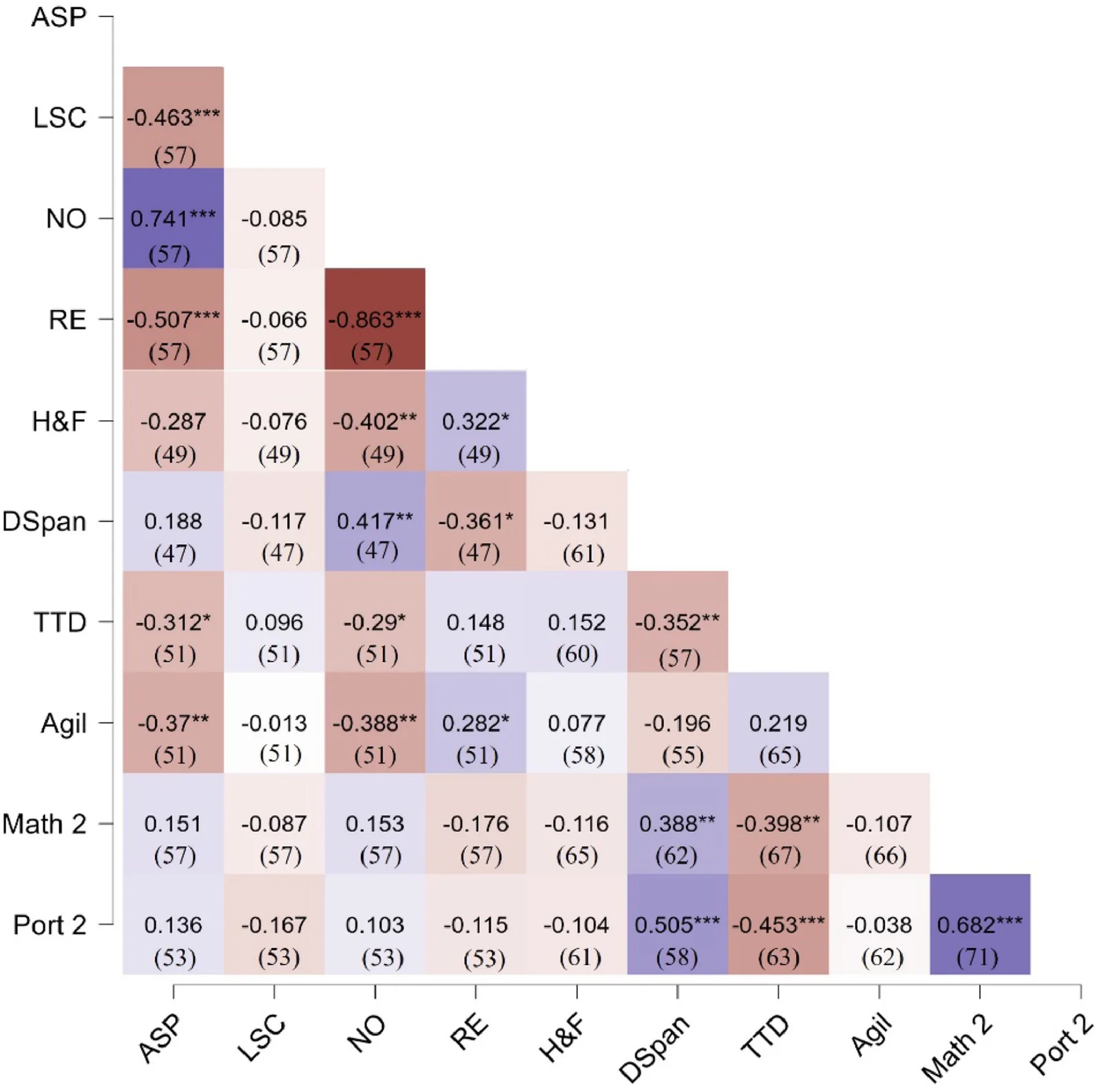
Heat map of correlations between the study variables. ASP, Average Shortest Path; LSC, Largest Strongly Connected; NO, Number of Nodes; RE, Repeated Edges; H&F, Hearts and Flowers; DSpan, Digit Span; TTD, Touch Test Disc; Agil, Agility; Math 2/Port 2 = Mathematics/Portuguese at Time 2. *p < .05, **p < .01, ***p < .001. Sample sizes in parentheses.

The scatter plots in Figure 2 demonstrated significant correlations between Digit Span and Touch Test Disc and performance in Portuguese and Mathematics. Digit Span showed significant positive correlations with Portuguese (r = 0.505; p < 0.001 - Panel A) and Mathematics (r = 0.388; p = 0.002 - Panel B), indicating that better working memory performance is associated with better academic grades. On the other hand, the Touch Test Disc showed significant negative correlations with Portuguese (r = −0.453; p < 0.001 - Panel C) and Mathematics (r = −0.398; p = 0.001 - Panel D), demonstrating that shorter test execution time (better motor performance) is similarly associated with better academic results.

**Figure 2:**
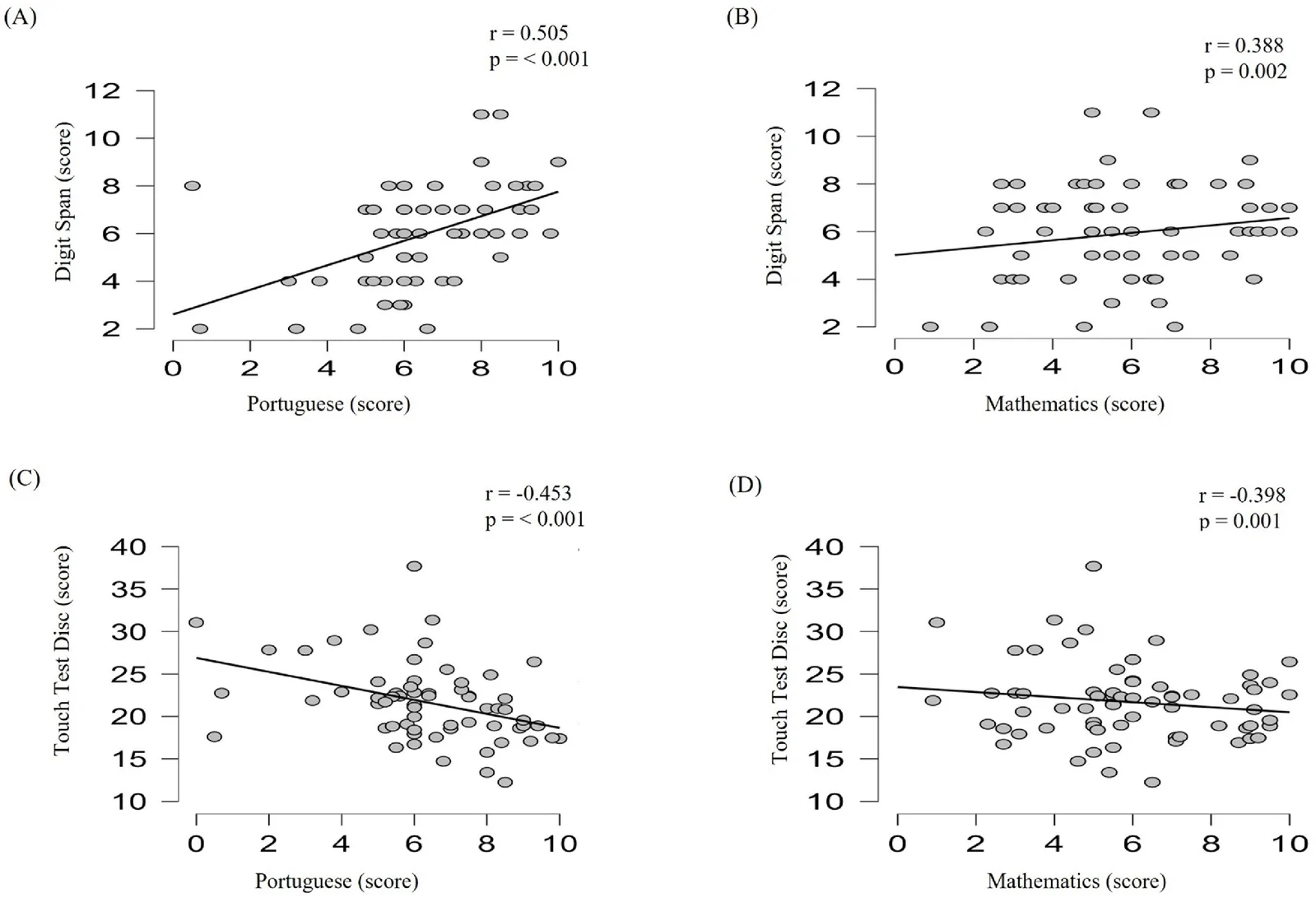
Relationships between predictor variables and academic performance at T2: (A) Digit Span × Portuguese (r = 0.505; p < 0.001), indicating that higher working memory scores are associated with better grades; (B) Digit Span × Mathematics (r = 0.388; p = 0.002), showing a similar positive association; (C) Touch Test Disc × Portuguese (r = −0.453; p < 0.001), where a shorter completion time (better motor performance) is associated with higher grades; (D) Touch Test Disc × Mathematics (r = −0.398; p = 0.001), indicating the same inverse relationship.

Table 2 presents the results of multiple linear regression analyses predicting performance in Portuguese and Mathematics at T2. For Portuguese performance, Digit Span emerged as a significant predictor (β = 0.492; p < 0.001), explaining 24.2% of the variance. For Mathematics performance, both age (β = −0.501; p < 0.001) and Digit Span (β = 0.413; p = 0.002) were significant predictors, together explaining 27.4% of the variance.

**Table 2:** Linear regression analysis for the prediction of academic performance at T2.

Dependent Variable	Predictor	B	SE	Β	t	p	R²	F
**Portuguese T2**	Digit Span	0.455	0.113	0.492	4.034	<0.001	0.242	F(1,51) = 16.272***
					
**Mathematics T2**	Age	−0.646	0.160	−0.501	−4.037	<0.001	0.274	F(2,54) = 10.190***
	Digit Span	0.445	0.134	0.413	3.325	0.002		
					

*Note: B, unstandardized coefficient; SE, standard error; β, standardized coefficient. ** p < 0.001.

## Discussion

The present study aimed to investigate cognitive, linguistic, and motor correlates and predictors of academic performance in children from Brazilian public schools. The results revealed that, among the assessed components of executive functions, specifically working memory, emerged as a significant predictor of academic performance, while motor coordination showed substantial associations but did not emerge as an independent predictor in the regression analyses. Digit Span emerged as the best predictor, explaining a meaningful portion of the variance in both Portuguese Language and Mathematics performance. This finding is consistent with a robust body of evidence positioning working memory as a central pillar for academic learning [25]. A noteworthy aspect of the present results was the magnitude of the correlation between Digit Span and Portuguese Language performance (r = 0.505), which surpassed the correlation with Mathematics (r = 0.388). This pattern contrasts with part of the literature, which often demonstrates stronger correlations between working memory and mathematics, given the need to maintain and manipulate numbers and mental operations [26]. This apparent contradiction may, in fact, reveal particularities of the present sample and socio-educational context. In environments of greater socioeconomic vulnerability, such as the one investigated, working memory may become an even more critical cognitive resource for text comprehension and the acquisition of written language skills that constitute the foundation for learning across all academic areas [27].

A noteworthy finding was the significant correlation observed between the Touch Test Disc (TTD) and academic performance. This result aligns with the premise that motor development is an inseparable component of cognition [7]. The TTD, by requiring planning, motor sequencing, and fine eye-hand coordination, acts as a mirror of the efficiency of brain networks that are equally recruited in academic tasks [28]. The observed negative correlation, where a shorter execution time (better motor performance) is associated with higher grades, suggests that eye-hand proficiency may facilitate performance in domains that rely on symbolic representation and spatial organization, such as mathematics, and graphomotor fluency, such as writing [17]. However, the regression analyses showed that the TTD did not independently predict academic performance after accounting for working memory and age. This suggests that the link between motor coordination and school grades may be explained by shared cognitive processes, particularly working memory, rather than a direct effect of motor skills on learning. The interpretation extends beyond a simple neural correlation. Children with better motor coordination, as measured by the TTD, likely not only possess more efficient neural systems but also engage more frequently and successfully in play and physical activities that require precision. This creates a virtuous cycle where motor competence facilitates participation, stimulating cognitive development and executive functions, ultimately resulting in better school performance [11].

In contrast to the cognitive predictors, neither the measure of inhibitory control nor the oral discourse metrics derived from non-semantic graph analysis showed the expected predictive power in the regression models. This result diverges from previous studies that found significant associations between discourse organization and academic outcomes [5]. The authors speculate that a possible explanation for this divergence may lie in the sociolinguistic characteristics of the studied population, since reading comprehension — which is closely related to oral discourse — is influenced by working memory and socioeconomic factors [4]. The picture description task may not have captured the complex discourse skills most relevant for written language, unlike free narrative or story retelling tasks. This limitation may have weakened the relationship the authors observed. Regarding inhibitory control, the null finding may reflect either that this skill is domain-specific (helping only in certain tasks rather than general academic performance), or that the Hearts and Flowers task overlap heavily with working memory in this age group [23]. Because both tasks require holding rules in mind and planning responses, their scores are strongly correlated. Once working memory is accounted for in the regression model, little unique variance from inhibitory control remains to predict academic outcomes.

Beyond the primary correlations with academic performance, an interesting pattern emerged in the analysis of speech graph metrics with other variables. The authors observed that better performance on the agility test was correlated with a lower number of Nodes (NO), implying a structurally smaller and less lexically diverse speech graph. Although not the primary focus, this may suggest a component of verbal impulsivity in children with greater motor speed. Conversely, and replicating previous findings, [29] a higher score on Digit Span was positively correlated with greater lexical diversity (NO) and negatively with word repetition (RE). This indicates that a better ability to hold and manipulate verbal information is associated with more elaborate and less repetitive speech. These secondary associations reinforce that the structure of spontaneous discourse captures fundamental cognitive components and pave the way for future investigations into how different executive functions are distinctly reflected in oral narrative.

It is crucial to recognize the study's limitations. First, the loss of about half of the original sample due to missing data may have introduced selection bias, limiting how well these results generalize. Second, the present sample came from a single region and consisted of children who volunteered for a physical activity intervention, so they may represent a specific subgroup of the population. Finally, the lack of direct measures of vocabulary or phonological awareness prevents us from fully exploring the linguistic mechanisms involved. Future research, with larger and more diverse samples and a more comprehensive battery of EF tasks, should investigate the specific neurocognitive mechanisms linking working memory and eye-hand coordination to different academic disciplines, as well as test the effectiveness of interventions that integrate executive function training with complex motor activities, such as capoeira, [30] in promoting school success. The present study aimed to investigate cognitive, linguistic, and motor correlates and predictors of academic performance in children from Brazilian public schools.

The results show that working memory, a specific component of executive functions, is a significant predictor of the academic performance of public-school children. While the Touch Test Disc (TTD) showed a significant inverse correlation with academic grades, suggesting that better eye-hand coordination is associated with higher performance, it did not emerge as an independent predictor in the regression models. These findings show that, in the present sample, working memory was the main cognitive skill driving academic success. They also warn us not to overstate the role of motor coordination based only on correlations. Future studies should address key conceptual and methodological gaps, including the addition of direct measures of cognitive flexibility, mediational models to test causal pathways, and more comprehensive language and discourse tasks. The simple nature of the instruments suggests they may be useful for research purposes, but further validation in diverse samples is needed before practical applications can be recommended.

## Funding

This work was supported by the research project "Motiveducação - Physical exercise for child development", selected under Call No 19/2023 from the Ministry of Sports of Brazil, through the National Council for Scientific and Technological Development (CNPq) (Grant No 445078/2023-5). The funders had no role in the study design, data collection and analysis, decision to publish, or preparation of the manuscript.

## Conflicts of interest

The authors declare no conflicts of interest.

## Data Availability

The data that support the findings of this study are available from the corresponding author.
